# 4-(2-Hy­droxy­eth­oxy)phenol

**DOI:** 10.1107/S1600536813019818

**Published:** 2013-07-24

**Authors:** Anne C. Meister, Mathias Lang, Martin Nieger, Stefan Bräse

**Affiliations:** a Institute of Organic Chemistry, Karlsruhe Institute of Technology, Fritz-Haber-Weg 6, 76131 Karlsruhe, Germany; b Laboratory of Inorganic Chemistry, Department of Chemistry, University of Helsinki, PO Box 55, 00014 University of Helsinki, Finland; c Institute of Organic Chemistry, Institute of Toxicology and Genetics, Karlsruhe Institute of Technology, Fritz-Haber-Weg 6, 76131 Karlsruhe, Germany

## Abstract

The asymmetric unit of the title compound, C_8_H_10_O_3_, contains four mol­ecules, which differ in the orientation of the hy­­droxy­ethyl group [O—C—C—O torsion angles = −168.89 (17), 72.9 (2), −65.8 (2) and 71.8 (2)°], as well as the orientation of the hy­droxy H atoms. Furthermore, the crystal structure displays two different types of strong hydrogen bond. The first is between an alcohol O—H and another alcohol O atom, and the second between an alcohol O—H group and an ether O atom. Additional weak hydrogen bonds between C—H groups and ether O atoms stabilize the structure.

## Related literature

For the synthesis of the title compound, see: Read & Miller (1932 ▶). For its biological activity, see: Smit *et al.* (1992 ▶). For its use in the synthesis of biologically active materials, see: Ding *et al.* (2009 ▶); Pitterna *et al.* (2004 ▶); Petrović & Brückner (2011 ▶). For its application in polymer synthesis, see: Nakano *et al.* (2000 ▶); Kaneda *et al.* (2004 ▶); Xi *et al.* (2010 ▶). For its use as a substrate for dye synthesis, see: Kelly (1996 ▶). For information about the cuprate, used for synthesis, see: Normant *et al.* (1980 ▶). For its reactivity, see: Semmelhack *et al.* (1985 ▶).
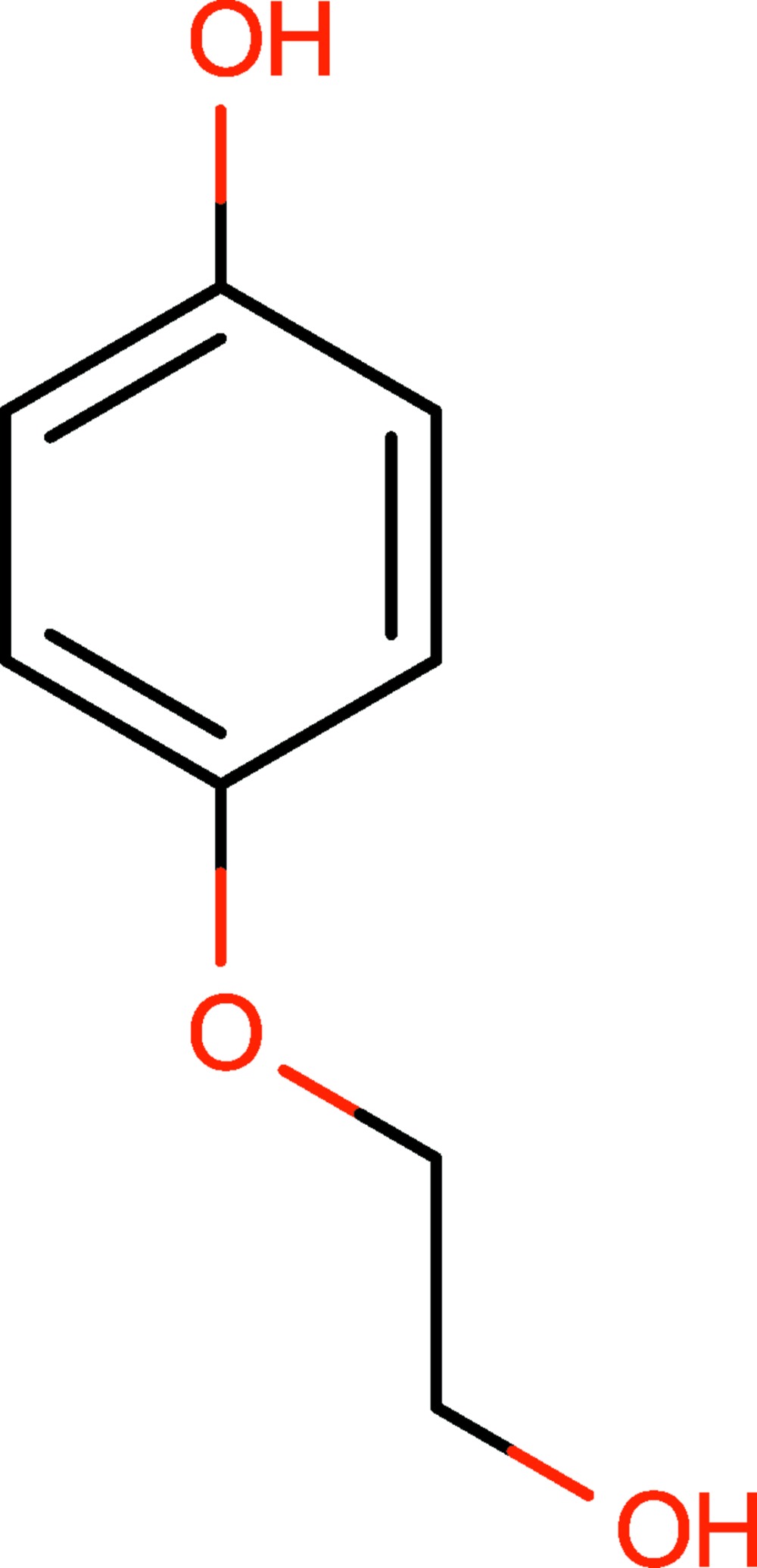


## Experimental

### 

#### Crystal data

C_8_H_10_O_3_
*M* *_r_* = 154.16
Triclinic, 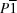

*a* = 10.0388 (10) Å
*b* = 10.2425 (8) Å
*c* = 15.0692 (11) Å
α = 83.916 (8)°
β = 86.470 (9)°
γ = 77.124 (8)°
*V* = 1500.8 (2) Å^3^
*Z* = 8
Mo *K*α radiation
μ = 0.10 mm^−1^
*T* = 123 K
0.16 × 0.08 × 0.04 mm

#### Data collection

Bruker Nonius KappaCCD diffractometer
Absorption correction: multi-scan (*SADABS*; Sheldrick, 2003 ▶) *T*_min_ = 0.910, *T*_max_ = 0.997
17940 measured reflections
5282 independent reflections
3204 reflections with *I* > 2σ(*I*)
*R*_int_ = 0.058

#### Refinement

*R*[*F* ^2^ > 2σ(*F* ^2^)] = 0.051
*wR*(*F* ^2^) = 0.110
*S* = 1.02
5282 reflections
421 parameters
48 restraints
H atoms treated by a mixture of independent and constrained refinement
Δρ_max_ = 0.21 e Å^−3^
Δρ_min_ = −0.27 e Å^−3^

### 

Data collection: *COLLECT* (Nonius, 1999 ▶); cell refinement: *EVALCCD* (Duisenberg *et al.*, 2003 ▶); data reduction: *EVALCCD*; program(s) used to solve structure: *SHELXS97* (Sheldrick, 2008 ▶); program(s) used to refine structure: *SHELXL97* (Sheldrick, 2008 ▶); molecular graphics: *SHELXTL* (Sheldrick, 2008 ▶); software used to prepare material for publication: *SHELXL97* and *publCIF* (Westrip, 2010 ▶).

## Supplementary Material

Crystal structure: contains datablock(s) I, global. DOI: 10.1107/S1600536813019818/mw2112sup1.cif

Structure factors: contains datablock(s) I. DOI: 10.1107/S1600536813019818/mw2112Isup2.hkl

Supplementary material file. DOI: 10.1107/S1600536813019818/mw2112Isup3.cml

Additional supplementary materials:  crystallographic information; 3D view; checkCIF report

## Figures and Tables

**Table 1:** Selected torsion angles (°)

O1*A*—C2*A*—C3*A*—O4*A*	−168.89 (17)
O1*B*—C2*B*—C3*B*—O4*B*	72.9 (2)
O1*C*—C2*C*—C3*C*—O4*C*	−65.8 (2)
O1*D*—C2*D*—C3*D*—O4*D*	71.8 (2)

**Table 2:** Hydrogen-bond geometry (Å, °)

*D*—H⋯*A*	*D*—H	H⋯*A*	*D*⋯*A*	*D*—H⋯*A*
O1*A*—H1*A*⋯O1*C* ^i^	0.84 (1)	1.85 (1)	2.671 (2)	165 (2)
O8*A*—H8*A*⋯O1*B* ^ii^	0.84 (1)	1.74 (1)	2.565 (2)	170 (2)
O1*B*—H1*B*⋯O1*D* ^iii^	0.84 (1)	1.88 (1)	2.707 (2)	168 (2)
O8*B*—H8*B*⋯O1*A* ^i^	0.84 (1)	1.79 (1)	2.617 (2)	169 (2)
O1*C*—H1*C*⋯O4*D*	0.84 (1)	2.12 (1)	2.830 (2)	142 (2)
O8*C*—H8*C*⋯O8*B* ^iv^	0.84 (1)	1.88 (1)	2.721 (2)	176 (2)
O1*D*—H1*D*⋯O8*C* ^iii^	0.84 (1)	2.02 (1)	2.800 (2)	155 (2)
O8*D*—H8*D*⋯O8*A* ^v^	0.84 (1)	1.88 (1)	2.707 (2)	167 (2)
C2*A*—H2*A*1⋯O4*B*	0.99	2.48	3.452 (3)	168
C2*B*—H2*B*2⋯O4*A*	0.99	2.58	3.450 (3)	147
C2*C*—H2*C*1⋯O8*A* ^vi^	0.99	2.44	3.402 (3)	163
C9*B*—H9*B*⋯O4*A* ^vii^	0.95	2.54	3.361 (3)	145
C2*C*—H2*C*2⋯O8*D* ^viii^	0.99	2.49	3.337 (3)	144
C2*D*—H2*D*2⋯O4*C*	0.99	2.57	3.478 (3)	152

Symmetry codes: (i) 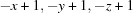; (ii) 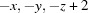; (iii) 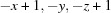; (iv) 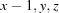; (v) 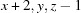; (vi) 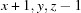; (vii) 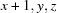; (viii) 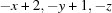.

**Fig. 1.**
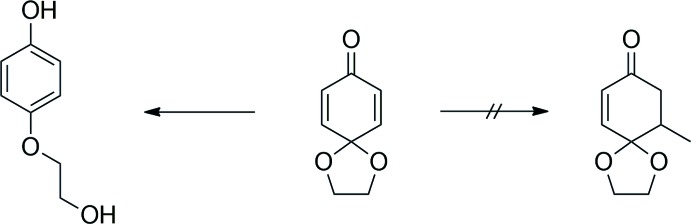
Unexpected synthesis of title compound. Reagents and conditions: a) CuBr*SMe2, MeMgCl, SMe2, THF, -20°C to -50°C, 15 h, 78%.

**Fig. 2.**
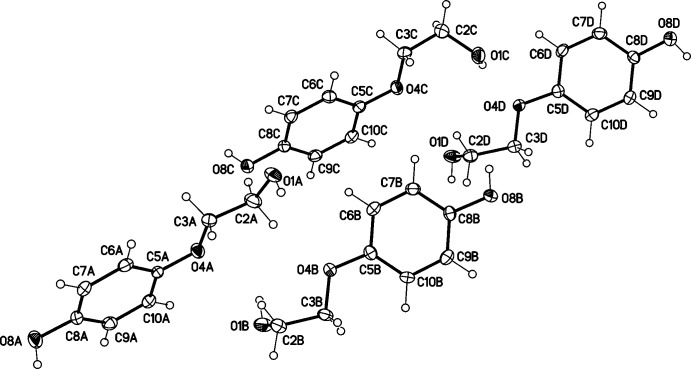
Content of the asymmetric unit showing all four crystallographic independent molecules (displacement parameters are drawn at 50% probability level).

**Fig. 3.**
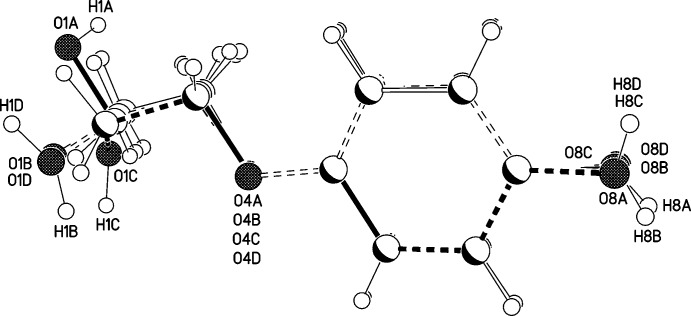
Least-squares fit of the four crystallographic independent molecules (fitted atoms O4, O8 and the C atoms of the phenyl ring).

**Fig. 4.**
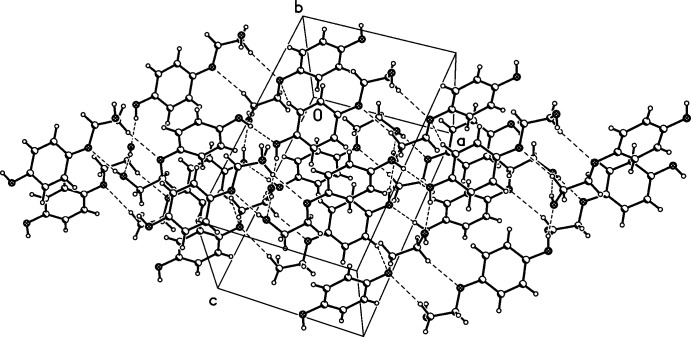
Packing diagram showing the strong and weak hydrogen bonds.

## supplementary crystallographic information

### Comment

Although the synthesis of the title compound, Scheme 1, has been known for about 80 years (Read & Miller, 1932), its crystal structure has never been reported. It has been used in syntheses of biologically active materials such as anti-cancer agents (Ding *et al.*, 2009) as it is toxic to melanoma cells (Smit *et al.*, 1992). It has also been used for the synthesis of an acaricide and insecticide substance (Pitterna *et al.*, 2004) and for the synthesis of steroid precursors (Petrović & Brückner, 2011). Further uses are as a monomer in polymer synthesis including liquid crystalline polymers (Nakano *et al.*, 2000) and coating rubbers (Kaneda *et al.*, 2004), in the synthesis of a surface active piperazine derivative (Xi *et al.*, 2010) and as a starting material for the synthesis of liquid-crystalline dyes (Kelly, 1996). We intended to do a 1,4 addition of a Normant cuprate (Normant *et al.*, 1980) to 1,4-dioxaspiro[4.5]deca-6,9-dien-8-one, but under the conditions applied we observed the title compound as the only product. A similar observation has been made (Semmelhack *et al.*, 1985) in the reaction of the same quinone with *n*BuLi. The intended synthesis along with the actual reaction is shown in Fig. 1. Fig. 2 shows the asymmetric unit with four independent molecules. They mainly differ in the orientation of the hydroxyethyl chain as well as in the orientation of the alcohol H atoms. The least-squares fit (Fig. 3) shows that molecules B and D are very similar and only differ in the orientation of the alcohol-H. Molecule C is similar to B, but the orientation of O1 differs. Molecule A shows a very different orientation of O1 (see also Table 1). Fig. 4 illustrates the packing including all hydrogen bonds. There is no obvious pattern, but there are different types of hydrogen bond resulting in different orientations of the H atoms. The strong hydrogen bonds O—H···O can be divided into those where the second oxygen is part of another alcohol function and those where the second oxygen is part of an ether function. Furthermore, there are also weak C—H···O hydrogen bonds.

### Experimental

The title compound was synthesized unplanned by the following procedure. A solution of 678 mg CuBr*SMe_2_ (3.30 mmol, 1.10 eq.) in 7 ml of dimethyl sulfide and 15 ml of ABS.THF was added at -30°C to a solution of 2.20 ml of methyl magnesium chloride (3 *M* in THF, 494 mg, 6.60 ml, 2.20 eq.) and the mixture was stirred at this temperature for 1 h. Then the mixture was cooled to -50°C and a solution of 460 mg 1,4-dioxaspiro[4.5]deca-6,9-dien-8-one (3.00 mmol, 1.00 eq.) in 5 ml of ABS.THF was added slowly to this mixture. Stirring was continued at this temperature for 15 h, then 11 ml of saturated ammonium chloride solution were added and the mixture was warmed to room temperature. Then air was bubbled through the solution for 1 h, the phases were separated and the aqueous phase was extracted with ethyl acetate (4 × 10 ml). The combined organic layers were dried over sodium sulfate and the solvent was removed under reduced pressure. The residue was purified by column chromatography (cyclohexane/ethyl acetate = 5:1) to yield the product as colorless crystals (361 mg, 2.34 mmol, 78%). The product crystallized from ethyl acetate after column chromatography by evaporation of the solvent with a rotavapor. m.p. = 90°C. The intended synthesis and the obtained product are shown in Fig. 1.

### Refinement

All H atoms were located in a difference electron density map. H atoms bound to carbon were refined using a riding model with aromatic C—H = 0.95 Å, secondary C—H = 0.99 Å, and with *U_iso_*(H) = 1.2*U_eq_*(C). The coordinates of the hydroxyl H atoms were refined freely with O—H distance retraints (0.84 (1) Å) and *U_iso_*(H) = 1.5*U_eq_*(O). Additionally 1,2 and 1,3 distance restraints (SADI) were used for the refinement of the hydroxyl group (for O—H and C—H(O) distance).

### Crystal data

**Table:**

C_8_H_10_O_3_	*Z* = 8
*M**_r_* = 154.16	*F*(000) = 656
Triclinic, *P*1	*D*_x_ = 1.365 Mg m^−^^3^
Hall symbol: -P 1	Mo *K*α radiation, λ = 0.71073 Å
*a* = 10.0388 (10) Å	Cell parameters from 109 reflections
*b* = 10.2425 (8) Å	θ = 1–25°
*c* = 15.0692 (11) Å	µ = 0.10 mm^−^^1^
α = 83.916 (8)°	*T* = 123 K
β = 86.470 (9)°	Plate, colourless
γ = 77.124 (8)°	0.16 × 0.08 × 0.04 mm
*V* = 1500.8 (2) Å^3^	

### Data collection

**Table:**

Bruker Nonius KappaCCD diffractometer	5282 independent reflections
Radiation source: fine-focus sealed tube	3204 reflections with *I* > 2σ(*I*)
Graphite monochromator	*R*_int_ = 0.058
rotation in φ and ω, 2° scans	θ_max_ = 25.0°, θ_min_ = 3.1°
Absorption correction: multi-scan (*SADABS*; Sheldrick, 2003)	*h* = −11→11
*T*_min_ = 0.910, *T*_max_ = 0.997	*k* = −12→12
17940 measured reflections	*l* = −17→17

### Refinement

**Table:**

Refinement on *F*^2^	Primary atom site location: structure-invariant direct methods
Least-squares matrix: full	Secondary atom site location: difference Fourier map
*R*[*F*^2^ > 2σ(*F*^2^)] = 0.051	Hydrogen site location: inferred from neighbouring sites
*wR*(*F*^2^) = 0.110	H atoms treated by a mixture of independent and constrained refinement
*S* = 1.02	*w* = 1/[σ^2^(*F*_o_^2^) + (0.0433*P*)^2^ + 0.2157*P*] where *P* = (*F*_o_^2^ + 2*F*_c_^2^)/3
5282 reflections	(Δ/σ)_max_ < 0.001
421 parameters	Δρ_max_ = 0.21 e Å^−^^3^
48 restraints	Δρ_min_ = −0.27 e Å^−^^3^

### Special details

**Table:**

Experimental. NMR spectra were recorded on a Bruker AM 400 spectrometer as solutions. Chemical shifts are expressed in parts per million (p.p.m., δ) downfield from tetramethylsilane (TMS) and are referenced to residual solvent peaks. The descriptions of signals include: m = multiplet, m_c_ = centered multiplet, bs = broad singlet. The spectra were analyzed as first order patterns. The signal structure in the ^13^C NMR was analyzed by DEPT and is described as follows: + = primary or tertiary C-atom (positive DEPT signal), – = secondary C-atom (negative DEPT signal) and C_q_ = quaternary C-atom (no DEPT signal). MS(EI) (electron impact mass spectrometry) was performed by using a FINNIGAN MAT 90 (70 eV). The mass peak [*M*]^+^ and characteristic fragment peaks are given as mass to charge ratio (m/*z*) and the intensity of the signals were indicated in percent, relative to the intensity of the base signal (100%). IR (infrared spectroscopy) was recorded on a FT–IR Bruker alpha and intensities of the signals are characterized as follows: *vs* (very strong, 0–10% transmission), s (strong, 11–30% transmission), m (medium, 31–70% transmission), w (weak, 71–90% transmission) and vw (very weak, 91–100% transmission). Solvents, reagents and chemicals were purchased from Aldrich, Acros and Merck. All solvents, reagents and chemicals were used as purchased. *R*_f_ (cyclohexane/ethyl acetate = 1:1) = 0.30. – ^1^H NMR (400 MHz, acetone-D_6_): δ/p.p.m. = 3.80–3.84 (m, 2H, 2 × C*H*_2_), 3.92–3.97 (m, 3H, O*H*, 2 × C*H*_2_), 6.76 (m_c_, 4H, 4 × C*H*_Ar_), 7.89 (bs, 1H, O*H*_Ar_). – ^13^C NMR (100 MHz, acetone-D_6_): δ /p.p.m. = 61.51 (–, *C*H_2_), 71.09 (–, *C*H_2_), 116.36 (+, 2 × *C*H_Ar_), 116.59 (+, 2 × *C*H_Ar_), 152.21 (C_q_, *C*_Ar_), 153.30 (C_q_, *C*_Ar_). – IR (ATR) ν/cm^-1^ = 3468 (vw), 3250 (w), 2927 (w), 1862 (vw), 1604 (vw), 1505 (*m*), 1448 (*m*), 1367 (w), 1298 (w), 1274 (vw), 1215 (*m*), 1173 (w), 1073 (w), 1051 (*m*), 900 (w), 884 (w), 824 (*m*), 766 (w), 750 (*m*), 708 (w), 660 (w), 638 (w), 565 (w), 525 (w). – MS (70 eV, EI): m/*z* (%) = 154 (42) [*M*]^+^, 126 (55) [*M* – C_2_H_4_]^+^, 110 (100) [*M* – C_2_H_4_O]^+^, 98 (29) [*M* – C_3_H_4_O]^+^, 43 (24) [C_2_H_3_O]^+^. – HR-EIMS (C_8_H_10_O_3_): calc. 154.0630; found 154.0631.
Geometry. All e.s.d.'s (except the e.s.d. in the dihedral angle between two l.s. planes) are estimated using the full covariance matrix. The cell e.s.d.'s are taken into account individually in the estimation of e.s.d.'s in distances, angles and torsion angles; correlations between e.s.d.'s in cell parameters are only used when they are defined by crystal symmetry. An approximate (isotropic) treatment of cell e.s.d.'s is used for estimating e.s.d.'s involving l.s. planes.
Refinement. Refinement of *F*^2^ against ALL reflections. The weighted *R*-factor *wR* and goodness of fit *S* are based on *F*^2^, conventional *R*-factors *R* are based on *F*, with *F* set to zero for negative *F*^2^. The threshold expression of *F*^2^ > σ(*F*^2^) is used only for calculating *R*-factors(gt) *etc*. and is not relevant to the choice of reflections for refinement. *R*-factors based on *F*^2^ are statistically about twice as large as those based on *F*, and *R*-factors based on ALL data will be even larger.

### Fractional atomic coordinates and isotropic or equivalent isotropic displacement parameters (Å^2^)

**Table:**

	*x*	*y*	*z*	*U*_iso_*/*U*_eq_	
O1A	0.18232 (16)	0.52473 (17)	0.64327 (11)	0.0282 (4)	
H1A	0.216 (2)	0.551 (2)	0.6858 (11)	0.042*	
C2A	0.1518 (2)	0.3973 (2)	0.67249 (16)	0.0259 (6)	
H2A1	0.2346	0.3354	0.6968	0.031*	
H2A2	0.1242	0.3581	0.6211	0.031*	
C3A	0.0380 (2)	0.4116 (2)	0.74358 (16)	0.0203 (6)	
H3A1	0.0568	0.4653	0.7904	0.024*	
H3A2	−0.0503	0.4569	0.7172	0.024*	
O4A	0.03354 (14)	0.27818 (15)	0.78052 (10)	0.0209 (4)	
C5A	−0.0668 (2)	0.2645 (2)	0.84542 (15)	0.0163 (5)	
C6A	−0.1655 (2)	0.3708 (2)	0.87354 (15)	0.0193 (6)	
H6A	−0.1661	0.4602	0.8488	0.023*	
C7A	−0.2637 (2)	0.3459 (2)	0.93823 (16)	0.0208 (6)	
H7A	−0.3316	0.4186	0.9578	0.025*	
C8A	−0.2632 (2)	0.2165 (2)	0.97428 (16)	0.0207 (6)	
O8A	−0.36470 (16)	0.19848 (17)	1.03765 (12)	0.0330 (5)	
H8A	−0.338 (2)	0.1330 (19)	1.0753 (13)	0.050*	
C9A	−0.1640 (2)	0.1099 (2)	0.94606 (16)	0.0207 (6)	
H9A	−0.1636	0.0206	0.9709	0.025*	
C10A	−0.0659 (2)	0.1338 (2)	0.88185 (15)	0.0188 (6)	
H10A	0.0022	0.0609	0.8626	0.023*	
O1B	0.31123 (17)	−0.00352 (17)	0.83922 (11)	0.0301 (4)	
H1B	0.279 (2)	0.009 (2)	0.7885 (9)	0.045*	
C2B	0.3478 (2)	0.1151 (2)	0.86098 (16)	0.0226 (6)	
H2B1	0.3557	0.1102	0.9265	0.027*	
H2B2	0.2740	0.1937	0.8434	0.027*	
C3B	0.4795 (2)	0.1358 (2)	0.81603 (15)	0.0214 (6)	
H3B1	0.5118	0.2062	0.8435	0.026*	
H3B2	0.5502	0.0513	0.8230	0.026*	
O4B	0.45661 (14)	0.17600 (15)	0.72330 (10)	0.0218 (4)	
C5B	0.5635 (2)	0.2121 (2)	0.67196 (15)	0.0177 (5)	
C6B	0.5347 (2)	0.2712 (2)	0.58594 (15)	0.0191 (6)	
H6B	0.4449	0.2840	0.5651	0.023*	
C7B	0.6359 (2)	0.3114 (2)	0.53055 (16)	0.0199 (6)	
H7B	0.6156	0.3520	0.4719	0.024*	
C8B	0.7674 (2)	0.2924 (2)	0.56081 (15)	0.0175 (5)	
O8B	0.87364 (15)	0.33208 (16)	0.50961 (11)	0.0225 (4)	
H8B	0.8447 (19)	0.377 (2)	0.4622 (10)	0.034*	
C9B	0.7968 (2)	0.2323 (2)	0.64565 (16)	0.0207 (6)	
H9B	0.8870	0.2189	0.6660	0.025*	
C10B	0.6956 (2)	0.1913 (2)	0.70164 (15)	0.0191 (6)	
H10B	0.7166	0.1493	0.7599	0.023*	
O1C	0.73642 (15)	0.41867 (16)	0.20251 (11)	0.0263 (4)	
H1C	0.7721 (19)	0.3400 (12)	0.1917 (16)	0.040*	
C2C	0.5993 (2)	0.4495 (3)	0.17389 (16)	0.0269 (6)	
H2C1	0.5892	0.3843	0.1321	0.032*	
H2C2	0.5800	0.5404	0.1412	0.032*	
C3C	0.4975 (2)	0.4452 (2)	0.25053 (16)	0.0228 (6)	
H3C1	0.5111	0.5047	0.2953	0.027*	
H3C2	0.4033	0.4765	0.2291	0.027*	
O4C	0.51734 (14)	0.30920 (15)	0.28973 (10)	0.0213 (4)	
C5C	0.4156 (2)	0.2782 (2)	0.34923 (15)	0.0177 (5)	
C6C	0.3025 (2)	0.3728 (2)	0.37613 (15)	0.0186 (6)	
H6C	0.2935	0.4653	0.3560	0.022*	
C7C	0.2028 (2)	0.3308 (2)	0.43256 (15)	0.0188 (6)	
H7C	0.1246	0.3950	0.4505	0.023*	
C8C	0.2159 (2)	0.1967 (2)	0.46305 (15)	0.0182 (6)	
O8C	0.11772 (15)	0.15193 (16)	0.51912 (11)	0.0246 (4)	
H8C	0.0441 (13)	0.2101 (17)	0.5174 (15)	0.037*	
C9C	0.3306 (2)	0.1033 (2)	0.43747 (15)	0.0202 (6)	
H9C	0.3405	0.0112	0.4590	0.024*	
C10C	0.4306 (2)	0.1434 (2)	0.38082 (15)	0.0199 (6)	
H10C	0.5092	0.0792	0.3636	0.024*	
O1D	0.80068 (17)	0.00017 (16)	0.32015 (11)	0.0271 (4)	
H1D	0.826 (2)	−0.0651 (14)	0.3584 (12)	0.041*	
C2D	0.8360 (2)	0.1160 (2)	0.34876 (16)	0.0207 (6)	
H2D1	0.8426	0.1046	0.4145	0.025*	
H2D2	0.7626	0.1959	0.3333	0.025*	
C3D	0.9696 (2)	0.1395 (2)	0.30650 (15)	0.0207 (6)	
H3D1	1.0007	0.2075	0.3373	0.025*	
H3D2	1.0403	0.0548	0.3121	0.025*	
O4D	0.95064 (14)	0.18600 (15)	0.21384 (10)	0.0201 (4)	
C5D	1.0629 (2)	0.2190 (2)	0.16558 (15)	0.0175 (5)	
C6D	1.0385 (2)	0.2832 (2)	0.08097 (15)	0.0209 (6)	
H6D	0.9484	0.3029	0.0596	0.025*	
C7D	1.1431 (2)	0.3191 (2)	0.02700 (16)	0.0223 (6)	
H7D	1.1251	0.3631	−0.0311	0.027*	
C8D	1.2757 (2)	0.2905 (2)	0.05823 (15)	0.0176 (5)	
O8D	1.37587 (15)	0.32883 (16)	0.00195 (11)	0.0257 (4)	
H8D	1.4524 (11)	0.288 (2)	0.0213 (14)	0.039*	
C9D	1.2997 (2)	0.2284 (2)	0.14293 (15)	0.0191 (6)	
H9D	1.3896	0.2098	0.1646	0.023*	
C10D	1.1939 (2)	0.1924 (2)	0.19739 (16)	0.0206 (6)	
H10D	1.2114	0.1499	0.2560	0.025*	

### Atomic displacement parameters (Å^2^)

**Table:**

	*U*^11^	*U*^22^	*U*^33^	*U*^12^	*U*^13^	*U*^23^
O1A	0.0300 (10)	0.0323 (10)	0.0228 (11)	−0.0131 (8)	−0.0012 (8)	0.0089 (8)
C2A	0.0302 (14)	0.0234 (14)	0.0219 (15)	−0.0051 (11)	0.0016 (11)	0.0046 (11)
C3A	0.0208 (13)	0.0185 (13)	0.0206 (14)	−0.0037 (10)	−0.0015 (10)	0.0024 (11)
O4A	0.0198 (9)	0.0179 (9)	0.0233 (10)	−0.0031 (7)	0.0032 (7)	0.0018 (7)
C5A	0.0132 (12)	0.0233 (14)	0.0131 (14)	−0.0055 (10)	−0.0020 (10)	−0.0005 (11)
C6A	0.0186 (12)	0.0178 (13)	0.0212 (15)	−0.0036 (10)	−0.0059 (11)	0.0022 (11)
C7A	0.0155 (12)	0.0214 (13)	0.0235 (15)	0.0000 (10)	−0.0027 (11)	−0.0006 (11)
C8A	0.0148 (12)	0.0262 (14)	0.0196 (14)	−0.0042 (11)	0.0008 (10)	0.0041 (11)
O8A	0.0236 (9)	0.0325 (11)	0.0343 (12)	0.0017 (8)	0.0085 (8)	0.0140 (8)
C9A	0.0217 (13)	0.0180 (13)	0.0223 (15)	−0.0060 (11)	−0.0043 (11)	0.0036 (11)
C10A	0.0164 (12)	0.0175 (13)	0.0211 (15)	−0.0003 (10)	−0.0029 (10)	−0.0018 (11)
O1B	0.0397 (10)	0.0335 (10)	0.0218 (11)	−0.0192 (8)	−0.0083 (8)	0.0046 (8)
C2B	0.0277 (14)	0.0236 (14)	0.0178 (14)	−0.0088 (11)	−0.0004 (11)	−0.0008 (11)
C3B	0.0244 (13)	0.0253 (14)	0.0130 (14)	−0.0039 (11)	−0.0005 (10)	0.0015 (11)
O4B	0.0172 (8)	0.0294 (9)	0.0177 (10)	−0.0054 (7)	0.0008 (7)	0.0025 (8)
C5B	0.0191 (12)	0.0172 (12)	0.0171 (14)	−0.0043 (10)	0.0032 (10)	−0.0048 (10)
C6B	0.0160 (12)	0.0190 (13)	0.0214 (15)	−0.0007 (10)	−0.0036 (11)	−0.0025 (11)
C7B	0.0215 (13)	0.0210 (13)	0.0159 (14)	−0.0025 (11)	−0.0021 (11)	0.0007 (11)
C8B	0.0169 (12)	0.0164 (12)	0.0201 (15)	−0.0056 (10)	0.0019 (11)	−0.0037 (10)
O8B	0.0196 (9)	0.0256 (10)	0.0202 (10)	−0.0041 (7)	−0.0007 (7)	0.0066 (8)
C9B	0.0159 (12)	0.0194 (13)	0.0257 (16)	−0.0004 (10)	−0.0036 (11)	−0.0026 (11)
C10B	0.0221 (13)	0.0210 (13)	0.0132 (14)	−0.0044 (11)	−0.0021 (10)	0.0021 (10)
O1C	0.0190 (9)	0.0250 (10)	0.0335 (11)	−0.0036 (8)	0.0029 (7)	−0.0008 (8)
C2C	0.0246 (14)	0.0344 (15)	0.0221 (15)	−0.0097 (12)	−0.0024 (11)	0.0032 (12)
C3C	0.0200 (13)	0.0184 (13)	0.0279 (15)	−0.0028 (11)	−0.0039 (11)	0.0068 (11)
O4C	0.0173 (8)	0.0208 (9)	0.0245 (10)	−0.0045 (7)	0.0030 (7)	0.0017 (7)
C5C	0.0174 (12)	0.0211 (13)	0.0158 (14)	−0.0058 (10)	−0.0043 (10)	−0.0015 (10)
C6C	0.0210 (13)	0.0160 (13)	0.0187 (14)	−0.0038 (10)	−0.0019 (11)	−0.0004 (10)
C7C	0.0160 (12)	0.0210 (13)	0.0179 (14)	0.0002 (10)	−0.0010 (10)	−0.0036 (11)
C8C	0.0174 (12)	0.0225 (13)	0.0153 (14)	−0.0064 (11)	−0.0020 (10)	0.0010 (11)
O8C	0.0187 (9)	0.0259 (10)	0.0253 (10)	−0.0011 (7)	0.0024 (8)	0.0058 (8)
C9C	0.0212 (13)	0.0164 (13)	0.0218 (15)	−0.0026 (10)	−0.0045 (11)	0.0025 (11)
C10C	0.0168 (12)	0.0200 (13)	0.0214 (15)	0.0002 (10)	−0.0033 (10)	−0.0029 (11)
O1D	0.0384 (10)	0.0211 (9)	0.0242 (11)	−0.0129 (8)	−0.0085 (8)	0.0046 (8)
C2D	0.0239 (13)	0.0176 (13)	0.0206 (14)	−0.0040 (10)	−0.0029 (11)	−0.0012 (11)
C3D	0.0206 (13)	0.0225 (13)	0.0186 (15)	−0.0040 (11)	−0.0031 (10)	0.0002 (11)
O4D	0.0160 (8)	0.0276 (9)	0.0160 (10)	−0.0046 (7)	−0.0014 (7)	0.0013 (7)
C5D	0.0186 (12)	0.0168 (12)	0.0183 (14)	−0.0051 (10)	−0.0008 (10)	−0.0037 (11)
C6D	0.0141 (12)	0.0253 (14)	0.0218 (15)	−0.0008 (11)	−0.0031 (11)	−0.0021 (11)
C7D	0.0247 (13)	0.0247 (14)	0.0164 (14)	−0.0038 (11)	−0.0025 (11)	0.0012 (11)
C8D	0.0175 (12)	0.0164 (12)	0.0177 (14)	−0.0021 (10)	0.0036 (10)	−0.0021 (10)
O8D	0.0192 (9)	0.0295 (10)	0.0271 (11)	−0.0056 (8)	−0.0005 (8)	0.0027 (8)
C9D	0.0145 (12)	0.0203 (13)	0.0222 (15)	−0.0023 (10)	−0.0017 (10)	−0.0037 (11)
C10D	0.0198 (13)	0.0226 (13)	0.0193 (15)	−0.0037 (11)	−0.0042 (11)	−0.0014 (11)

### Geometric parameters (Å, º)

**Table:**

O1A—C2A	1.425 (3)	O1C—C2C	1.425 (3)
O1A—H1A	0.840 (9)	O1C—H1C	0.835 (9)
C2A—C3A	1.511 (3)	C2C—C3C	1.498 (3)
C2A—H2A1	0.9900	C2C—H2C1	0.9900
C2A—H2A2	0.9900	C2C—H2C2	0.9900
C3A—O4A	1.429 (3)	C3C—O4C	1.431 (3)
C3A—H3A1	0.9900	C3C—H3C1	0.9900
C3A—H3A2	0.9900	C3C—H3C2	0.9900
O4A—C5A	1.379 (3)	O4C—C5C	1.385 (3)
C5A—C6A	1.383 (3)	C5C—C6C	1.389 (3)
C5A—C10A	1.391 (3)	C5C—C10C	1.390 (3)
C6A—C7A	1.388 (3)	C6C—C7C	1.385 (3)
C6A—H6A	0.9500	C6C—H6C	0.9500
C7A—C8A	1.377 (3)	C7C—C8C	1.382 (3)
C7A—H7A	0.9500	C7C—H7C	0.9500
C8A—O8A	1.382 (3)	C8C—O8C	1.382 (3)
C8A—C9A	1.388 (3)	C8C—C9C	1.387 (3)
O8A—H8A	0.838 (9)	O8C—H8C	0.840 (9)
C9A—C10A	1.381 (3)	C9C—C10C	1.382 (3)
C9A—H9A	0.9500	C9C—H9C	0.9500
C10A—H10A	0.9500	C10C—H10C	0.9500
O1B—C2B	1.420 (3)	O1D—C2D	1.426 (3)
O1B—H1B	0.836 (9)	O1D—H1D	0.840 (9)
C2B—C3B	1.495 (3)	C2D—C3D	1.506 (3)
C2B—H2B1	0.9900	C2D—H2D1	0.9900
C2B—H2B2	0.9900	C2D—H2D2	0.9900
C3B—O4B	1.432 (3)	C3D—O4D	1.437 (3)
C3B—H3B1	0.9900	C3D—H3D1	0.9900
C3B—H3B2	0.9900	C3D—H3D2	0.9900
O4B—C5B	1.379 (3)	O4D—C5D	1.389 (3)
C5B—C10B	1.390 (3)	C5D—C6D	1.381 (3)
C5B—C6B	1.390 (3)	C5D—C10D	1.387 (3)
C6B—C7B	1.381 (3)	C6D—C7D	1.379 (3)
C6B—H6B	0.9500	C6D—H6D	0.9500
C7B—C8B	1.389 (3)	C7D—C8D	1.398 (3)
C7B—H7B	0.9500	C7D—H7D	0.9500
C8B—C9B	1.378 (3)	C8D—O8D	1.373 (3)
C8B—O8B	1.387 (3)	C8D—C9D	1.374 (3)
O8B—H8B	0.839 (8)	O8D—H8D	0.841 (9)
C9B—C10B	1.389 (3)	C9D—C10D	1.393 (3)
C9B—H9B	0.9500	C9D—H9D	0.9500
C10B—H10B	0.9500	C10D—H10D	0.9500
			
C2A—O1A—H1A	108.1 (14)	C2C—O1C—H1C	108.1 (14)
O1A—C2A—C3A	110.68 (19)	O1C—C2C—C3C	112.20 (19)
O1A—C2A—H2A1	109.5	O1C—C2C—H2C1	109.2
C3A—C2A—H2A1	109.5	C3C—C2C—H2C1	109.2
O1A—C2A—H2A2	109.5	O1C—C2C—H2C2	109.2
C3A—C2A—H2A2	109.5	C3C—C2C—H2C2	109.2
H2A1—C2A—H2A2	108.1	H2C1—C2C—H2C2	107.9
O4A—C3A—C2A	106.28 (18)	O4C—C3C—C2C	107.90 (19)
O4A—C3A—H3A1	110.5	O4C—C3C—H3C1	110.1
C2A—C3A—H3A1	110.5	C2C—C3C—H3C1	110.1
O4A—C3A—H3A2	110.5	O4C—C3C—H3C2	110.1
C2A—C3A—H3A2	110.5	C2C—C3C—H3C2	110.1
H3A1—C3A—H3A2	108.7	H3C1—C3C—H3C2	108.4
C5A—O4A—C3A	117.24 (17)	C5C—O4C—C3C	116.79 (17)
O4A—C5A—C6A	124.0 (2)	O4C—C5C—C6C	123.7 (2)
O4A—C5A—C10A	115.83 (19)	O4C—C5C—C10C	116.1 (2)
C6A—C5A—C10A	120.2 (2)	C6C—C5C—C10C	120.3 (2)
C5A—C6A—C7A	119.5 (2)	C7C—C6C—C5C	119.3 (2)
C5A—C6A—H6A	120.3	C7C—C6C—H6C	120.3
C7A—C6A—H6A	120.3	C5C—C6C—H6C	120.3
C8A—C7A—C6A	120.5 (2)	C8C—C7C—C6C	120.7 (2)
C8A—C7A—H7A	119.8	C8C—C7C—H7C	119.7
C6A—C7A—H7A	119.8	C6C—C7C—H7C	119.7
C7A—C8A—O8A	117.6 (2)	C7C—C8C—O8C	121.9 (2)
C7A—C8A—C9A	120.0 (2)	C7C—C8C—C9C	119.6 (2)
O8A—C8A—C9A	122.4 (2)	O8C—C8C—C9C	118.4 (2)
C8A—O8A—H8A	111.9 (15)	C8C—O8C—H8C	109.9 (13)
C10A—C9A—C8A	119.9 (2)	C10C—C9C—C8C	120.4 (2)
C10A—C9A—H9A	120.0	C10C—C9C—H9C	119.8
C8A—C9A—H9A	120.0	C8C—C9C—H9C	119.8
C9A—C10A—C5A	120.0 (2)	C9C—C10C—C5C	119.7 (2)
C9A—C10A—H10A	120.0	C9C—C10C—H10C	120.2
C5A—C10A—H10A	120.0	C5C—C10C—H10C	120.2
C2B—O1B—H1B	110.2 (14)	C2D—O1D—H1D	108.4 (14)
O1B—C2B—C3B	112.92 (19)	O1D—C2D—C3D	112.26 (19)
O1B—C2B—H2B1	109.0	O1D—C2D—H2D1	109.2
C3B—C2B—H2B1	109.0	C3D—C2D—H2D1	109.2
O1B—C2B—H2B2	109.0	O1D—C2D—H2D2	109.2
C3B—C2B—H2B2	109.0	C3D—C2D—H2D2	109.2
H2B1—C2B—H2B2	107.8	H2D1—C2D—H2D2	107.9
O4B—C3B—C2B	108.39 (18)	O4D—C3D—C2D	109.02 (18)
O4B—C3B—H3B1	110.0	O4D—C3D—H3D1	109.9
C2B—C3B—H3B1	110.0	C2D—C3D—H3D1	109.9
O4B—C3B—H3B2	110.0	O4D—C3D—H3D2	109.9
C2B—C3B—H3B2	110.0	C2D—C3D—H3D2	109.9
H3B1—C3B—H3B2	108.4	H3D1—C3D—H3D2	108.3
C5B—O4B—C3B	117.01 (17)	C5D—O4D—C3D	116.55 (16)
O4B—C5B—C10B	123.5 (2)	C6D—C5D—C10D	119.6 (2)
O4B—C5B—C6B	116.82 (19)	C6D—C5D—O4D	116.18 (19)
C10B—C5B—C6B	119.6 (2)	C10D—C5D—O4D	124.3 (2)
C7B—C6B—C5B	120.4 (2)	C7D—C6D—C5D	120.9 (2)
C7B—C6B—H6B	119.8	C7D—C6D—H6D	119.5
C5B—C6B—H6B	119.8	C5D—C6D—H6D	119.5
C6B—C7B—C8B	119.9 (2)	C6D—C7D—C8D	119.7 (2)
C6B—C7B—H7B	120.1	C6D—C7D—H7D	120.2
C8B—C7B—H7B	120.1	C8D—C7D—H7D	120.2
C9B—C8B—O8B	116.93 (19)	O8D—C8D—C9D	123.2 (2)
C9B—C8B—C7B	119.9 (2)	O8D—C8D—C7D	117.3 (2)
O8B—C8B—C7B	123.1 (2)	C9D—C8D—C7D	119.4 (2)
C8B—O8B—H8B	110.6 (13)	C8D—O8D—H8D	108.3 (13)
C8B—C9B—C10B	120.5 (2)	C8D—C9D—C10D	120.8 (2)
C8B—C9B—H9B	119.7	C8D—C9D—H9D	119.6
C10B—C9B—H9B	119.7	C10D—C9D—H9D	119.6
C9B—C10B—C5B	119.6 (2)	C5D—C10D—C9D	119.6 (2)
C9B—C10B—H10B	120.2	C5D—C10D—H10D	120.2
C5B—C10B—H10B	120.2	C9D—C10D—H10D	120.2
			
O1A—C2A—C3A—O4A	−168.89 (17)	O1C—C2C—C3C—O4C	−65.8 (2)
C2A—C3A—O4A—C5A	−177.71 (18)	C2C—C3C—O4C—C5C	−166.76 (18)
C3A—O4A—C5A—C6A	2.9 (3)	C3C—O4C—C5C—C6C	−4.2 (3)
C3A—O4A—C5A—C10A	−178.20 (19)	C3C—O4C—C5C—C10C	174.66 (19)
O4A—C5A—C6A—C7A	178.77 (19)	O4C—C5C—C6C—C7C	176.9 (2)
C10A—C5A—C6A—C7A	−0.1 (3)	C10C—C5C—C6C—C7C	−1.9 (3)
C5A—C6A—C7A—C8A	0.0 (3)	C5C—C6C—C7C—C8C	0.8 (3)
C6A—C7A—C8A—O8A	−179.3 (2)	C6C—C7C—C8C—O8C	−179.7 (2)
C6A—C7A—C8A—C9A	0.1 (3)	C6C—C7C—C8C—C9C	0.6 (3)
C7A—C8A—C9A—C10A	0.0 (3)	C7C—C8C—C9C—C10C	−0.9 (3)
O8A—C8A—C9A—C10A	179.3 (2)	O8C—C8C—C9C—C10C	179.4 (2)
C8A—C9A—C10A—C5A	−0.1 (3)	C8C—C9C—C10C—C5C	−0.2 (3)
O4A—C5A—C10A—C9A	−178.77 (19)	O4C—C5C—C10C—C9C	−177.3 (2)
C6A—C5A—C10A—C9A	0.2 (3)	C6C—C5C—C10C—C9C	1.6 (3)
O1B—C2B—C3B—O4B	72.9 (2)	O1D—C2D—C3D—O4D	71.8 (2)
C2B—C3B—O4B—C5B	174.15 (18)	C2D—C3D—O4D—C5D	176.98 (18)
C3B—O4B—C5B—C10B	10.5 (3)	C3D—O4D—C5D—C6D	−170.15 (19)
C3B—O4B—C5B—C6B	−170.01 (19)	C3D—O4D—C5D—C10D	9.3 (3)
O4B—C5B—C6B—C7B	179.37 (19)	C10D—C5D—C6D—C7D	1.2 (3)
C10B—C5B—C6B—C7B	−1.1 (3)	O4D—C5D—C6D—C7D	−179.3 (2)
C5B—C6B—C7B—C8B	0.2 (3)	C5D—C6D—C7D—C8D	−0.1 (3)
C6B—C7B—C8B—C9B	0.6 (3)	C6D—C7D—C8D—O8D	−179.8 (2)
C6B—C7B—C8B—O8B	−178.9 (2)	C6D—C7D—C8D—C9D	−0.8 (3)
O8B—C8B—C9B—C10B	179.1 (2)	O8D—C8D—C9D—C10D	179.6 (2)
C7B—C8B—C9B—C10B	−0.4 (3)	C7D—C8D—C9D—C10D	0.7 (3)
C8B—C9B—C10B—C5B	−0.5 (3)	C6D—C5D—C10D—C9D	−1.3 (3)
O4B—C5B—C10B—C9B	−179.2 (2)	O4D—C5D—C10D—C9D	179.2 (2)
C6B—C5B—C10B—C9B	1.3 (3)	C8D—C9D—C10D—C5D	0.3 (3)

### Hydrogen-bond geometry (Å, º)

**Table:**

*D*—H···*A*	*D*—H	H···*A*	*D*···*A*	*D*—H···*A*
O1*A*—H1*A*···O1*C*^i^	0.84 (1)	1.85 (1)	2.671 (2)	165 (2)
O8*A*—H8*A*···O1*B*^ii^	0.84 (1)	1.74 (1)	2.565 (2)	170 (2)
O1*B*—H1*B*···O1*D*^iii^	0.84 (1)	1.88 (1)	2.707 (2)	168 (2)
O8*B*—H8*B*···O1*A*^i^	0.84 (1)	1.79 (1)	2.617 (2)	169 (2)
O1*C*—H1*C*···O4*D*	0.84 (1)	2.12 (1)	2.830 (2)	142 (2)
O8*C*—H8*C*···O8*B*^iv^	0.84 (1)	1.88 (1)	2.721 (2)	176 (2)
O1*D*—H1*D*···O8*C*^iii^	0.84 (1)	2.02 (1)	2.800 (2)	155 (2)
O8*D*—H8*D*···O8*A*^v^	0.84 (1)	1.88 (1)	2.707 (2)	167 (2)
C2*A*—H2*A*1···O4*B*	0.99	2.48	3.452 (3)	168
C2*B*—H2*B*2···O4*A*	0.99	2.58	3.450 (3)	147
C2*C*—H2*C*1···O8*A*^vi^	0.99	2.44	3.402 (3)	163
C9*B*—H9*B*···O4*A*^vii^	0.95	2.54	3.361 (3)	145
C2*C*—H2*C*2···O8*D*^viii^	0.99	2.49	3.337 (3)	144
C2*D*—H2*D*2···O4*C*	0.99	2.57	3.478 (3)	152

Symmetry codes: (i) −*x*+1, −*y*+1, −*z*+1; (ii) −*x*, −*y*, −*z*+2; (iii) −*x*+1, −*y*, −*z*+1; (iv) *x*−1, *y*, *z*; (v) *x*+2, *y*, *z*−1; (vi) *x*+1, *y*, *z*−1; (vii) *x*+1, *y*, *z*; (viii) −*x*+2, −*y*+1, −*z*.
